# New Species, New Record, and Antagonistic Potential of *Torula* (*Torulaceae*, *Pleosporales*) from Jilin Province, China

**DOI:** 10.3390/microorganisms13071459

**Published:** 2025-06-23

**Authors:** Rong Xu, Yue Zhang, Wenxin Su, Yu Li

**Affiliations:** 1 School of Food Science and Engineering, Yangzhou University, Yangzhou 225127, China; zyxurong66@126.com; 2 Internationally Cooperative Research Center of China for New Germplasm Breeding of Edible Mushroom, Jilin Agricultural University, Changchun 130118, China; xrzhangyue66@163.com (Y.Z.); suwenxin1220@163.com (W.S.)

**Keywords:** antifungal activity, hyphomycetous fungi, northeastern China, phylogeny, taxonomy

## Abstract

During a survey of ascomycetous fungi associated with plant litter and submerged wood in Jilin Province, China, two hyphomycetous fungi were discovered. Morphological examination and molecular phylogenetic analyses revealed that these isolates represent two species within the genus *Torula*, which are herein described as *Torula changchunensis* sp. nov. and a new host record of *T. mackenziei*. Detailed morphological characteristics are provided, and the phylogenetic relationships of the new species are also discussed. The new species differs from *T. chinensis* and *T. phytolaccae* by having smaller conidiogenous cells, smaller conidia, and fewer septa. Preliminary assessments were conducted on *T. changchunensis* and *T. mackenziei* to evaluate their antagonistic activity against two pathogenic fungi (*Cladobotryum mycophilum* and *Botrytis cinerea*) and two pathogenic bacteria (*Staphylococcus aureus* and *Bacillus subtilis*). *Torula changchunensis* sp. nov. exhibited 67.18% inhibition against *C. mycophilum* and moderate inhibition against the pathogenic bacteria, whereas *Torula mackenziei* showed moderate-to-weak inhibitory activity against both pathogenic fungi and bacteria.

## 1. Introduction

The family *Torulaceae*, established by Corda and typified by *Torula* Pers. [1], represents a distinct lineage within the order *Pleosporales*. Currently, this family is known only from its asexual morphs, which are characterized by grayish-brown to black, superficial, effuse, and powdery colonies; micro- or macronematous conidiophores, with or without apical branches; doliiform to ellipsoid or clavate, brown, smooth to verruculose, mono- to polyblastic conidiogenous cells; and brown, monilioid, phragmosporous, acrogenous, smooth to verrucose conidia [2,3,4,5,6,7,8]. Members of *Torulaceae* are commonly associated with persistent woody stems as saprobes and are occasionally pathogenic on dicotyledons [6,7,8,9,10,11,12,13]. Crous et al. [6] investigated the classification of this family and recognized *Dendryphion* and *Torula* as belonging to *Torulaceae*. Subsequently, the genera *Cylindrotorula*, *Neotorula*, *Rostriconidium*, *Rutola*, and *Sporidesmioides* were also accommodated within the family based on morphological characteristics and phylogenetic analyses [5,7,14,15,16].

*Torula* was established by Persoon in 1795, with *T. herbarum* (=*T. monilis*) designated as the type species [17]. Species of *Torula* are defined by distinct asexual morphological features, such as monoblastic or polyblastic, as well as terminal or lateral conidiogenous cells with a thickened, heavily melanized basal wall and a thin-walled apex that frequently collapses and becomes coronate [3]. Crane and Schoknecht (1977) documented the conidiogenesis of *Torula* species using light and transmission electron microscopy [18]. Similar observations were reported by Mason, Hughes, Subramanian, and other researchers, who contributed significantly to the understanding of phenotypic variation among *Torula* species [19,20,21,22,23]. To date, more than 500 epithets are listed under *Torula* in Index Fungorum (http://www.indexfungorum.org/; accessed on 16 April 2025), with many species transferred to other genera. Unfortunately, there is a shortage of molecular information for most *Torula* species [6], and for many of them, recollection and culture isolation will be necessary.

Members of *Torula* typically inhabit terrestrial and aquatic environments in temperate to tropical regions as saprobes [24]. Several species also occur as plant pathogens or endophytes. For example, *T. herbarum* has been reported as the causal agent of stem blight on *Ziziphus mauritiana* (*Rhamnaceae*) [12] and was also isolated from the surface of apple fruit, where it forms part of a fungal complex responsible for apple sooty blotch [13]. Additionally, *T. herbarum* has been identified as an endophyte on the leaves of *Solanum nigrum* [25]. Some *Torula* species were also detected in the air of natural environments around crop fields after harvest [26]. Moreover, this genus has been documented to produce various interesting secondary metabolites, such as a novel dechlorinated aromatic lactone, herbarin, dehydroherbarin, and o-methylherbarin, and has exhibited antibacterial, antifungal, antiamoebic, and potentially anticancer activities [27,28,29,30,31,32].

During a survey of saprobic fungi in Jilin Province, China, two isolates were obtained from woody substrates. In this study, we introduce a new species (*Torula changchunensis*) and a new record of *T. mackenziei*, which were collected from submerged wood and *Xanthoceras sorbifolium* (*Sapindaceae*), respectively. The new taxon was compared morphologically with other *Torula* species. Multi-gene phylogenetic analyses based on ITS, LSU, SSU, *tef*1-α, and *rpb*2 loci were performed to determine the taxonomic placement of the novel species. In addition, we tested the antimicrobial activities of these fungi in vitro to screen for new biocontrol agents.

## 2. Materials and Methods

### 2.1. Sample Collection, Isolation, and Morphological Observation

Decaying branches of *X. sorbifolium* were collected from Jilin Agricultural University, and submerged wood was collected from Jingyuetan National Scenic Area in Changchun, Jilin Province. The specimens were transferred to the laboratory in sealed plastic bags with labels describing specific collection information. A Zeiss Stemi 2000C stereomicroscope (ZEISS, Oberkochen, Germany) fitted with a Leica DFC450C digital camera (Leica, Wetzlar, Germany) was used to examine the macromorphological sample characteristics of samples from the host. Colonies on the substrate surface were transferred to a temporary slide with sterile water. Morphological characteristics and microscopic measurements were performed using a Zeiss AX10 light microscope equipped with an Axiocam 506 digital camera (ZEISS, Oberkochen, Germany). The photos were processed using Adobe Photoshop CC2019 (Adobe Systems, San Jose, CA, USA). The fungi were obtained by single-spore isolation methods described by Senanayake et al. [33]. Germinated conidia were transferred aseptically to the new potato dextrose agar (PDA) and incubated at 25 °C in dark.

Specimens were deposited in the Herbarium of Mycology, Jilin Agricultural University (HMJAU), Changchun, China. Pure fungal cultures were deposited in the International Cooperation Research Center of China for New Germplasm Breeding of Edible Mushrooms Culture Collection (CCMJ) and the Engineering Research Center of Edible and Medicinal Fungi, Ministry of Education culture collection (EMFCC), Changchun, China. The new taxon was registered in Mycobank [34].

### 2.2. DNA Extraction, PCR Amplification, and Sequencing

After culturing the isolates in PDA at 25 °C for 2 weeks, the mycelium from the pure cultures was collected and flash-frozen with liquid nitrogen. Genomic DNA was extracted using a NuClean PlantGen DNA Kit (CWBIO, Taizhou, China), adhering to the instructions provided by the manufacturer. The internal transcribed spacer regions (ITS), large subunit (LSU) rDNA, small subunit (SSU) rDNA, translation elongation factor 1-alpha gene (*tef*1-α), and the RNA polymerase II second-largest subunit (*rpb*2) were amplified by polymerase chain reaction (PCR) using ITS5/ITS4 [35], LR0R/LR5 [36], NS1/NS4 [35], TEF1-983F/TEF1-2218R [37,38], and fRPB2-5F/fRPB2-7cR [39] primer pairs, respectively. The amplification reactions were performed using 20 μL PCR mixtures containing 9 μL of sterilized water, 10 μL of 2 × Es Taq MasterMix (Dye) (CWBIO, Taizhou, China), 0.3 μL of forward and reverse primers, and 0.4 μL of the DNA template. The amplification conditions were as follows: 94 °C for 5 min, then 35 cycles of denaturation at 94 °C for 30 s, annealing at 55 °C for 45 s, elongation at 72 °C for 90 s, and a final extension at 72 °C for 10 min. The PCR products were confirmed on 1% agarose electrophoresis gels stained with standard DNA dye. Purification and sequencing were conducted at Sangon Biotech Co. in Shanghai, China.

### 2.3. Phylogenetic Analysis

The preliminary identification of the new strains was carried out by using BLASTn searches (https://www.ncbi.nlm.nih.gov/, accessed on 18 June 2024), and the newly generated nucleotide sequence data were deposited in GenBank. Our newly obtained sequences, along with those from recent publications [11,16,40] and the GenBank database, used in the phylogenetic analysis, are displayed in Table 1. *Roussoella hysterioides* (CBS 125434 and CBS 546.94) was selected as the outgroup. The individual gene sequences were assembled using BioEdit version 7.2.5 [41], aligned with MAFFT version 7 (http://mafft.cbrc.jp/alignment/server/index.html, accessed on 6 February 2025), and adjusted manually in AliView [42,43]. The sequence datasets were combined using SequenceMatrix v.1.7.8 [44]. 

The phylogenetic trees were prepared using both maximum likelihood (ML) and Bayesian inference (BI) analyses. ML analysis was conducted using RAxML-HPC2 on XSEDE (version 8.2.12), accessible via the CIPRES web portal (http://www.phylo.org/portal2/, accessed on 10 February 2025) [45], with an RAxML rapid bootstrapping of 1000 replicates. The best-fit evolutionary models for both individual and combined datasets were calculated using the Akaike Information Criterion (AIC) with jModeltest 2.1.10 on the CIPRES web portal [46]. BI analyses were performed using MrBayes version 3.2.6 on the CIPRES web portal [47], with simultaneous Markov chains running for 5,000,000 generations and trees sampled every 100th generation. Finally, phylogenetic trees were illustrated in FigTree 1.4.3 [48] and edited using Microsoft Office PowerPoint. The ML bootstrap support values (ML, greater than or equal to 70%) and Bayesian posterior probability values (BPP, greater than or equal to 0.90) are shown on the branches at the nodes.

### 2.4. Preliminary Assessment of Antagonistic Effects on Pathogens

To determine the antimicrobial activity of *T. mackenziei* (CCMJ 13080) and *T. changchunensis* (EMFCC 0042), the pathogen isolates of *Cladobotryum mycophilum* (*C. mycophilum*), *Botrytis cinerea* (*B. cinerea*), *Staphylococcus aureus* (*S. aureus*), and *Bacillus subtilis* (*B. subtilis*) were obtained from CCMJ and used as indicator strains. The working cultures of these pathogens were prepared by transferring mycelium/bacteria colonies onto PDA and lysogeny broth (LB) plates and incubating them for 6 days at 25 °C and for 2 days at 30 °C, respectively.

Dual culture testing in vitro was carried out to screen the antagonism of *T. mackenziei* (CCMJ 13080) and *T. changchunensis* (EMFCC 0042) against the fungal pathogens [49]. A 6 mm fungal pathogen disk was placed on one side of a 9 cm diameter PDA plate. Meanwhile, a 6 mm antagonistic fungal disk was positioned in a symmetrical location on the opposite side of the pathogen disk, maintaining a distance of 3 cm from the pathogen and 2 cm from the edge of the PDA plate. Plates inoculated only with the cultures of fungal pathogens served as negative controls. The plates were incubated at 25 °C for one week. The assay was replicated three times. The radial growth of fungal colonies was documented, and the percentage of mycelial growth inhibition (PGI) was determined using the following formula: PGI (%) = (R1 − R2)/R1 × 100 [50], where R1 is the radial growth of the control pathogen and R2 is the radial growth of the pathogen in confrontation with each isolate. Statistical analysis of variance (ANOVA) and the least significant difference (LSD) test, where *p* ≤ 0.05, were performed using SPSS Statistics version 22 (SPSS, Inc., Chicago, IL, USA).

For evaluating the antagonism between pathogenic bacteria and indicator strains, we put 200 µL of bacterial suspensions with adjusted concentrations into the PDA medium, and then transferred stock agar plugs containing antagonistic fungus mycelium onto culture dishes containing pathogenic bacteria, inoculating each dish with four agar plugs. The control was placed on a filter paper in the center, and streptomycin sulfate (30 μg/mL) was dropped onto each filter paper. The assay was replicated three times, and observations of the inhibition zone were carried out after 24 h at 37 °C.

## 3. Results

### 3.1. Phylogenetic Analyses

**Figure 1:**
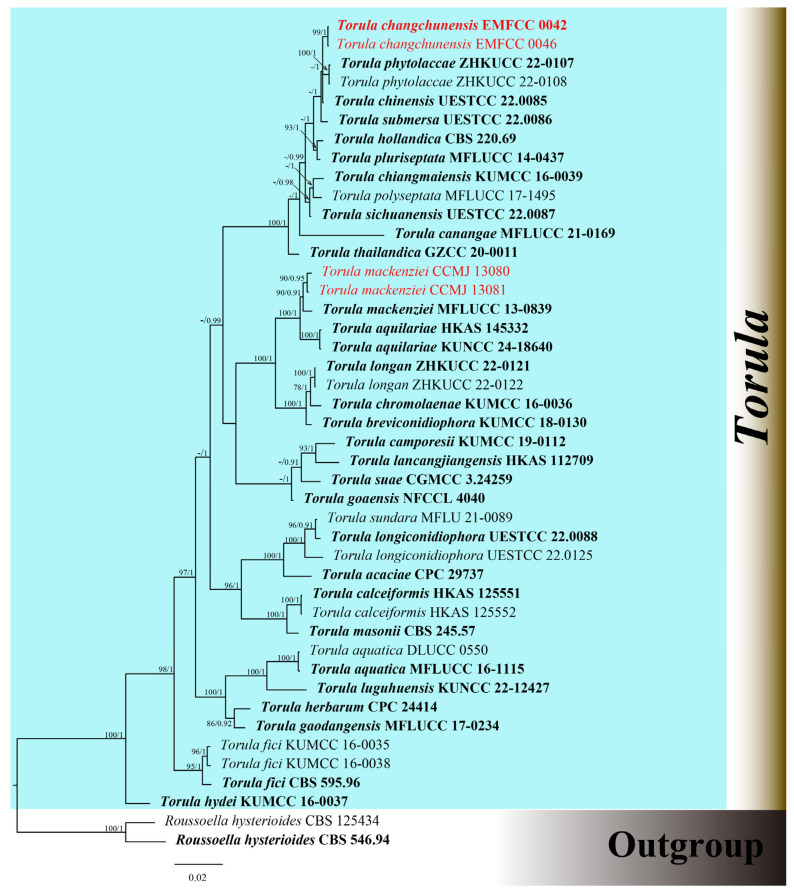
Phylogenetic tree of *Torula* generated from Bayesian inference analysis based on combined ITS, LSU, SSU, *tef*1-α, and *rpb*2 sequence data. Maximum likelihood bootstrap support values ≥ 70% (ML) and Bayesian posterior probabilities ≥ 0.90 (BPP) are given at the nodes as ML/BPP. The tree is rooted with *Roussoella hysterioides* (CBS 125434, CBS 546.94). The type strains are in bold, and the new strains are in red.

The combined dataset of ITS, LSU, SSU, *tef*1-α, and r*pb*2 genes comprised 41 strains, including our newly sequenced strains. Multi-locus sequences were concatenated, which comprised 4069 characters, including gaps (ITS: 1–514; LSU: 515–1346; SSU: 1347–2304; tef1-α: 2305–3128; rpb2: 3129–4069). The GTR + I + G was the best model used for the analysis of all datasets. The best-scoring RAxML tree had a final optimization likelihood value of −15947.007918. The matrix had 1036 distinct alignment patterns, with 23.14% of undetermined characters or gaps. Estimated base frequencies were as follows: A = 0.247649; C = 0.261367; G = 0.268353; and T = 0.222631. Substitution rates were AC = 2.079376; AG = 4.432116; AT = 1.800766; CG = 1.236905; CT = 10.709148; and GT = 1.000000. The proportion of invariable sites was I = 0.588501; the gamma distribution shape parameter was α = 0.440095. In the BI analysis, a total of 98 trees were sampled after the 20% burn-in phase, with a stop value of 0.008597. The topologies of ML and BI analysis were congruent (Figure 1 and Figure S1).

The phylogenetic results shown in Figure 1 demonstrated an undescribed species and one known species. Our strains of CCMJ 13080 and CCMJ 13081 grouped together with the ex-type strain of *T. mackenziei* (MFLUCC 13-0839) with a bootstrap support of 90% ML and 0.91 BPP. The newly described taxa *T. changchunensis* (EMFCC 0042, EMFCC 0046) formed an independent lineage and clustered with *T. phytolaccae* (ZHKUCC 22-0107, ZHKUCC 22-0108) and *T. chinensis* (UESTCC 22.0085) with 57% ML and 1.00 BPP support.

### 3.2. Taxonomy

**Figure 2:**
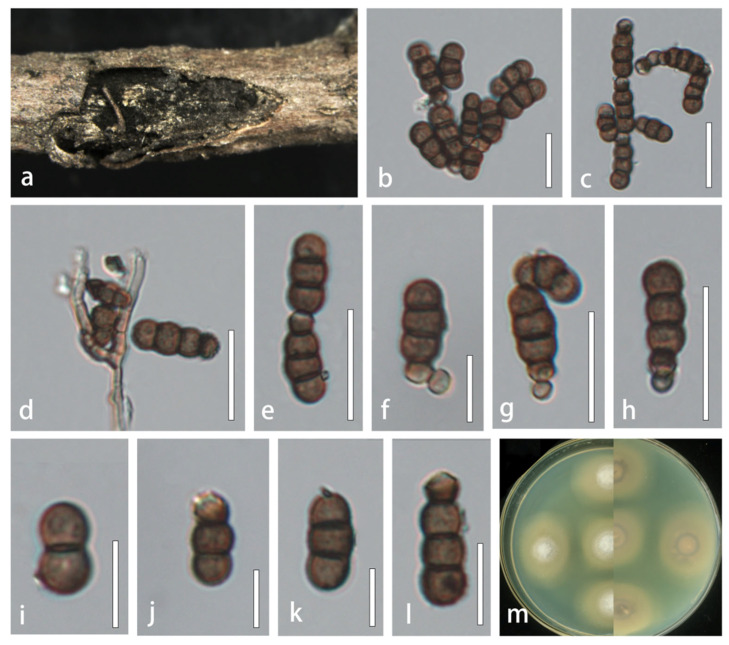
*Torula mackenziei* (HMJAU 64848). (**a**) Colonies on host substrate. (**b**,**c**) Conidia in catenated chain. (**d**) Conidiophores with conidiogenous cells. (**e**–**l**) Conidia. (**m**) Culture characteristics on PDA. Scale bars: (**a**) = 100 μm; (**b**−**e**,**g**−**h**) = 20 μm; (**f**,**i**−**l**) = 10 μm.

*Torula mackenziei* J.F. Li, Phookamsak & K.D. Hyde, Mycol. Progress 16 (4): 455 (2017), new host record and new geological record (Figure 2).

**Index Fungorum number**: IF819537; **Facesofungi number**: FoF 02714.

**Description.** *Saprobic* on decaying branches of *Xanthoceras sorbifolium*. **Sexual morph**: Undetermined. **Asexual morph**: Hyphomycetous. *Colonies* effuse, black, powdery on natural substrate. *Mycelium* immersed, composed of branched, smooth-walled, septate, light brown hyphae. *Conidiophores* 7–22 (−38) × 3–5 μm ($\bar{x}$ = 13 × 4 μm, *n* = 10), solitary, micronematous to semi-macronematous, mononematous, erect, minutely verruculose, without apical branches, thick-walled, sometimes reduced to conidiogenous cells, ellipsoid to subglobose, brown. *Conidiogenous cells* 4–9 × 3–6 μm ($\bar{x}$ = 7 × 4 μm, *n* = 10), globose to ellipsoid, integrated, blastic, terminal, paler at apex, smooth to minutely verruculose, brown, thick-walled. *Conidia* (5−) 11–23 × 4–9 μm ($\bar{x}$ = 14 × 6 μm, *n* = 50), yellowish brown to dark brown, acrogenous, phragmosporous, catenated, rounded at both ends, paler at apex, smooth to minutely verruculose, composed of globose to doliiform cells, 1–3-septate, constricted at septa, monilioid, with conidial chains in branches. *Conidial secession* schizolytic.

**Culture characteristics.** Colonies on PDA reaching 30 mm in diameter after 10 days at 25 °C. Cultures from above, dense, circular, cottony, covered with white, fluffy-like aerial mycelium, entire edges, paler yellowish, radiating outwardly. The reverse side is a pale pink in the center that gradually extends outwards, while the color changes to dark brown with creamy yellow.

**Material examined.** CHINA, Jilin Province, Changchun city, Jilin Agriculture University, on decaying branches of *Xanthoceras sorbifolium* (*Sapindaceae*), 17 June 2021, XR22.1 (HMJAU 64848), living culture CCMJ 13080; Siping city, forest farm in Lishu County, on decaying branches of *Xanthoceras sorbifolium* (*Sapindaceae*), 20 August 2021, Rong Xu, XR22.2 (HMJAU 64849), living culture CCMJ 13081.

**Distribution.** China, Thailand.

**Notes.** Li et al. [8] first reported *Torula mackenziei*, which was isolated from a dead branch of *Bidens pilosa* (*Asteraceae*) in Thailand. Subsequently, this species was also collected from submerged wood in southwest China, with the morphology characterized by larger conidiophores, conidiogenous cells, and conidia [16]. Our new isolates (HMJAU 64848 and HMJAU 64849) have similar morphologies to the holotype specimen of *T. mackenziei* (MFLU 16-2820). Pairwise sequence comparisons between the new isolates and the holotype revealed minor differences. In the multi-gene phylogenetic analyses, our strains (CCMJ 13080 and CCMJ 13081) derived from this study clustered with the other *T. mackenziei* strain (MFLUCC 13-0839) with high statistical support (90% ML, 0.91 BYPP). This is a new record of *T. mackenziei* collected from *Sapindaceae* in northeastern China.

**Figure 3:**
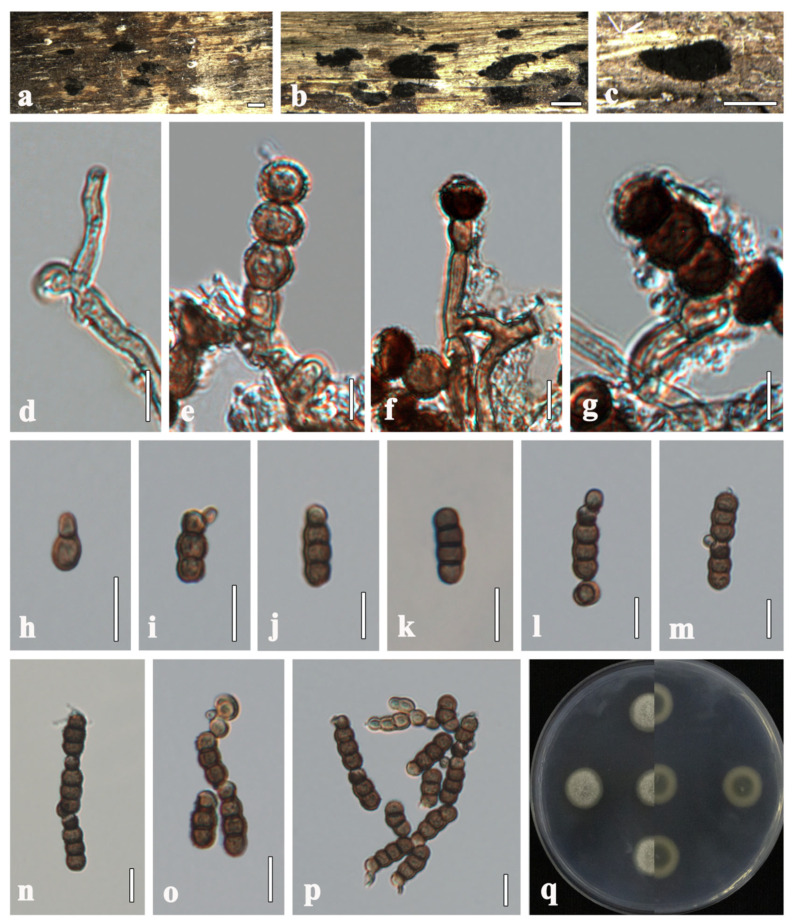
*Torula changchunensis* (HMJAU 64900, **holotype**). (**a**–**c**) Colonies on host substrate. (**d**–**g**) Conidiophores with conidiogenous cells. (**h**–**m**) Conidia. (**n**–**p**) Conidia in catenated chain. (**q**) Culture characteristics on PDA. Scale bars: (**a**−**c**) = 500 μm; (**d**−**g**) = 5 μm; (**h**−**p**) = 10 μm.

***Torula changchunensis*** R. Xu & Y. Li, sp. nov. (Figure 3)

**MycoBank number**: 856481

**Etymology**: Referring to Changchun city, where this fungus was collected.

**Description**. *Saprobic* on submerged wood in freshwater. **Sexual morph**: Undetermined. **Asexual morph**: Hyphomycetous. *Colonies* sporadic, black, effuse, dense, powdery. *Mycelium* immersed to superficial, composed of pale brown to brown, septate, branched, smooth-walled hyphae. *Conidiophores* 2–5 µm wide, macronematous to semi-macronematous, subcylindrical, straight or slightly flexuous, consisting of 1–3 cells, smooth-walled, pale brown to brown, sometimes reduced to conidiogenous cells, arising from lateral and terminal on a hypha. *Conidiogenous cells* 3–5 × 3–5 µm ($\bar{x}$ = 4 × 4, *n* = 20), monoblastic, lateral to terminal, thick-walled, cupulate to subglobose, dark brown, verruculose. *Conidia* 9–21 × 4–6 µm ($\bar{x}$ = 16 × 5, *n* = 40), solitary or in branched chains, acrogenous, phragmosporous, verruculose, straight or slightly curved, rounded at both ends, often paler at apex, thick-walled, composed of subglobose cells, 1–4-septate, constricted at the septa, pale brown to dark brown, monilioid. *Conidial secession* schizolytic.

**Culture characteristics**. Colonies on PDA reaching 20 mm in diameter after 8 days at 25 °C. Cultures from above, circular, cream to greenish-white, dense, entire edge, velvety, covered with fluffy-like aerial mycelium, umbonate, slightly radiating in the lower part; reverse orange at the center, light brown, radiating outwardly; reverse greenish gray at the center, white radiating outwardly, without pigment produced in PDA.

**Material examined**. CHINA, Jilin Province, Changchun city, Jinyue district, Jingyuetan national forest park, on submerged wood, 18 October 2023, Rong Xu, HO1 (HMJAU 64900, holotype), ex-type living culture EMFCC 0042; HO1b (HMJAU 70080, isotype), living culture EMFCC 0046.

**Notes**. In morphology, our new isolate matched with the generic concept of the genus *Torula* in having monoblastic, terminal, or lateral conidiogenous cells, and acropetal chains of dark phragmoconidia [3,51,52]. In the multi-gene phylogenetic analysis, *Torula changchunensis* is closely related to *T. chinensis* (UESTCC 22.0085) and *T. phytolaccae* (ZHKUCC 22-0107, ZHKUCC 22-0108). However, it differs from *T. chinensis* and *T. phytolaccae* by smaller conidiogenous cells (3–5 × 3–5 µm vs. 5–8 × 5–7 µm vs. 4–6.7 × 5–7.7 μm), smaller conidia (9–21 × 4–6 µm vs. 6–37 × 4–8 µm vs. (9–)20–34 (−63) × 5–9 µm), and fewer septa (1–4 vs. 1–7 vs. 2–12) [11,53].

In a BLASTn search of GenBank, the closest match to the ITS sequence had 98.11% similarity to *T. chinensis* (UESTCC 22.0085), and the closest match to the LSU sequence had 97.56% similarity to *T. herbarum* (CBS 246.57), while the *tef*1-α and *rpb*2 genes showed 96.23% and 98.68% similarity with *T. phytolaccae* (ZHKUCC 23-0884) and *T. phytolaccae* (ZHKUCC 22-0108), respectively. Therefore, we introduced *T. changchunensis* as a novel species based on the significant differences in morphology and phylogeny.

### 3.3. Preliminary Assessment of Antagonistic Activity on Pathogens

**Figure 4:**
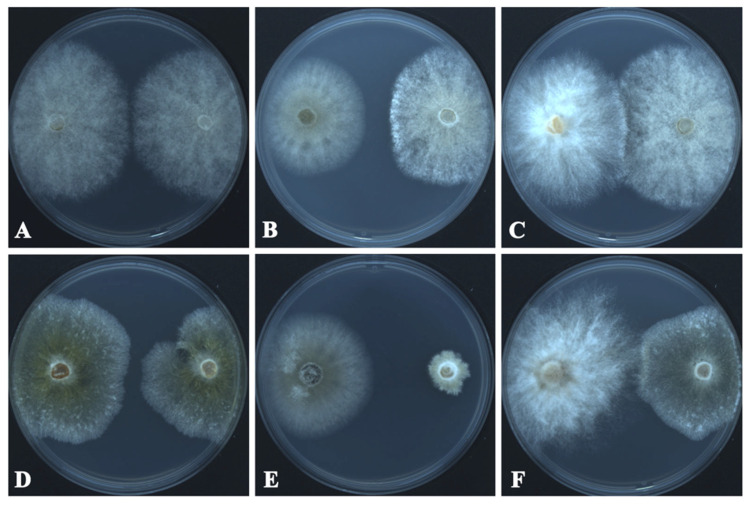
In vitro growth inhibition of *Torula* species (left colonies) against fungal pathogens (right colonies) 7 days after the start of the confrontation. (**A**) CK: *Botrytis cinerea* vs. *Bo. cinerea*. (**B**) *T. changchunensis* (EMFCC 0042) vs. *Bo. cinerea*. (**C**) *T. mackenziei* (CCMJ 13080) vs. *Bo. cinerea*. (**D**) Control Check (CK): *Cladobotryum mycophilum* vs. *C. mycophilum*. (**E**) *T. changchunensis* (EMFCC 0042) vs. *C. mycophilum*. (**F**) *T. mackenziei* (CCMJ 13080) vs. *C. mycophilum*.

The growth inhibition and radial growth of *B. cinerea* and *C. mycophilum* in the control and test Petri plates are shown in Table 2 and Figure 4. *T. changchunensis* (EMFCC 0042) demonstrated a stronger resistance to *C. mycophilum* compared to other strains. Specifically, *T. changchunensis* (EMFCC 0042) exhibited a potent inhibition effect against *C. mycophilum* at 67.18 ± 0.0169%, whereas its inhibition against *B. cinerea* was moderate (19.01 ± 0.0406%). In addition, the moderate inhibitory activity of *T. mackenziei* (CCMJ 13080) against *B. cinerea* and *C. mycophilum* was 10.20 ± 0.0182% and 24.57 ± 0.0259%, respectively (Table 2). 

**Figure 5:**
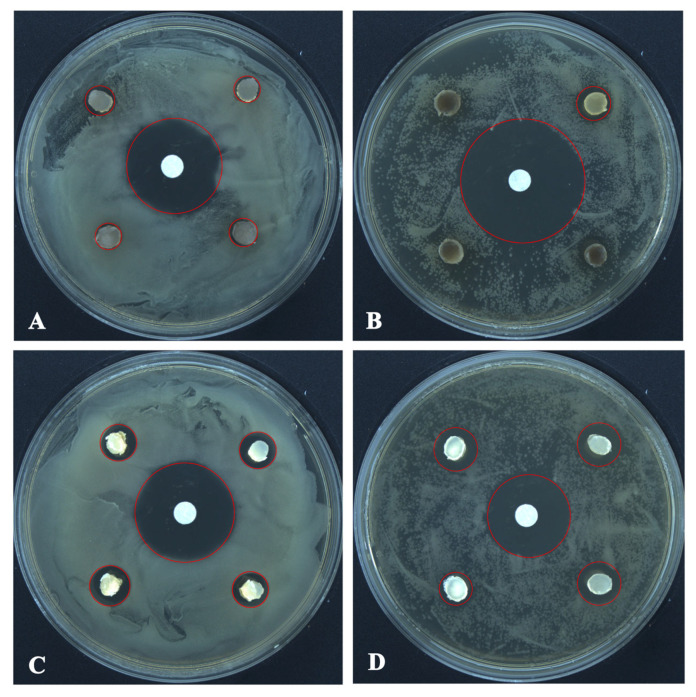
The antagonistic activity of *Torula* species against pathogenic bacteria 24 h after the start of the confrontation. (**A**) *T. mackenziei* (CCMJ 13080) vs. *S. aureus*. (**B**) *T. mackenziei* (CCMJ 13080) vs. *B. subtilis*. (**C**) *T. changchunensis* (EMFCC 0042) vs. *S. aureus*. (**D**) *T. changchunensis* (EMFCC 0042) vs. *B. subtilis*.

The antagonistic activity of *T*. *changchunensis* (EMFCC 0042) and *T. mackenziei* (CCMJ 13080) against two pathogenic bacteria is shown in Figure 5. The antagonistic reactions between *T*. *changchunensis* (EMFCC 0042) and *S. aureus* and *B. subtilis* were significant (11.79 ± 1.0496a, 13.57 ± 1.6085a). However, *T. mackenziei* (CCMJ 13080) exhibited barely any inhibition activity against the two tested pathogenic bacteria, and the diameters of the inhibition zones were 6.30 mm and 6.13 mm, respectively.

## 4. Discussion

Due to the lack of molecular data for most *Torula* species, taxonomic confusion persists within this genus; some species may in fact be conspecific or belong to other genera [11]. For example, *Torula rhombica* and *T. terrestris* were transferred to the genus *Bahusandhika* based on morphological characteristics by Crane and Miller [3]. He et al. considered *T. longiconidiophora* to be a synonym of *T. sundara* based on phylogenetic analysis, despite differences in conidial size [10]. Therefore, it is necessary to further investigate the taxonomic status of *Torula* epithets that were established prior to the advent of Sanger sequencing. With the development of molecular biology, relationships among *Torula* species have become clearer, and several new species have been identified through combined multi-locus phylogenetic analyses and morphological characterization [5,8,11,24,53,54,55,56]. In this study, we report two *Torula* species from northeastern China based on multi-locus phylogeny combined with morphological features. Phylogenetic analysis revealed that *Torula phytolaccae*, *T. chinensis*, and *T. changchunensis* cluster together in a subclade with 57% ML/1.00 BPP support. Although these species share some morphological similarities, *T. changchunensis* can be distinguished by its significantly smaller conidiogenous cells, smaller conidia, and fewer septa.

Jilin Province, located in northeastern China, belongs to the temperate climatic zone, which is characterized by harsh winters with minimum temperatures reaching as low as −20 °C [54]. Owing to its unique geographical location and climate, we discovered a novel *Torula* species on submerged wood in this region, consistent with the genus’s typical ecological preferences. In southern China, *Torula* species are also commonly found as saprobes in aquatic environments [57]. Our findings contribute to the known species diversity of *Torula* in China (Table 3) and emphasize the importance of microfungal diversity studies. Additionally, we report a new record of *T. mackenziei* from the decaying branches of *X. sorbifolium* (*Sapindaceae*), an endemic tree in northern China [58]. Previously, *T. mackenziei* was collected from *B. pilosa* in Chiang Rai, Thailand, and from submerged wood in Yunnan Province, China. These regions are located in tropical and subtropical zones, respectively, whereas Jilin Province lies in a temperate zone. According to earlier studies, members of *Torula* have been frequently associated with hosts from the family *Asteraceae* [8,56]. Our study extends the known host range of *T. mackenziei* to different environments, suggesting that northeastern China supports a broader diversity of these fungi.

The genus *Torula* has been shown to produce a wide array of secondary metabolites [27,28,29,30,31,32]. In this study, the antagonistic activity of two isolates against pathogens was evaluated in vitro. *Torula changchunensis* (EMFCC 0042) exhibited the strongest inhibitory effect against *C. mycophilum*, a causative agent of cobweb disease in several important mushroom crops [59,60]. Our results suggest that *T. changchunensis* holds promise as a biocontrol agent for the prevention and management of cobweb disease, contributing to organic, integrated, and sustainable mushroom cultivation practices. Moreover, since several *Torula* species have demonstrated antimicrobial properties [30,31], further investigation into the antimicrobial potential of additional *Torula* species would be valuable. However, in this study, only preliminary in vitro screening of antimicrobial activity was conducted. Future research should focus on compound extraction, the screening of secondary metabolites, and the elucidation of antagonistic mechanisms.

## Figures and Tables

**Table 1:** Taxa dataset used in the phylogenetic analyses with information on strain/isolate, host, and country. The ex-types are in bold, and the newly generated sequences are in blue.

Species	Strain/Isolate	Host	Country	GenBank Accession Numbers				
				ITS	LSU	SSU	*tef*1-α	*rpb*2
*Roussoella hysterioides*	CBS 546.94	-	-	KF443405	KF443381	AY642528	KF443399	KF443392
*R. hysterioides*	CBS 125434	-	Japan	MH863689	MH875155	-	-	-
***Torula acaciae***	**CPC 29737**	***Acacia koa***	**USA**	**KY173471**	**KY173560**	-	-	**KY173594**
*T. aquatica*	DLUCC 0550	submerged wood	China	MG208166	MG208145	-	MG207996	MG207976
***T. aquatica***	**MFLUCC 16-1115**	**submerged wood**	**China**	**MG208167**	**MG208146**	-	-	**MG207977**
***aquilariae***	**KUNCC 24-18640**	***Aquilaria sinensis***	**China**	**PQ788522**	**PQ788524**	-	**PQ810572**	**PQ810570**
***T. aquilariae***	**HKAS 145332**	***Aquilaria sinensis***	**China**	**PQ788521**	**PQ788523**	-	**PQ810571**	**PQ810569**
***T. breviconidiophora***	**KUMCC 18-0130**	decaying wood	Thailand	MK071670	MK071672	MK071697	MK077673	-
***T. calceiformis***	**HKAS 125551**	**dead wood**	**China**	**OP751054**	**OP751052**	**OP751050**	**OQ630512**	**OQ630510**
*T. calceiformis*	HKAS 125552	dead wood	China	OP751055	OP751053	OP751051	OQ630513	OQ630511
***T. camporesii***	**KUMCC 19-0112**	**herbaceous litter**	**China**	**MN507400**	**MN507402**	**MN507401**	**MN507403**	**MN507404**
***T. canangae***	**MFLUCC 21-0169**	***Cananga odorata***	**Thailand**	**OL966950**	**OL830816**	-	**ON032379**	-
***T. changchunensis***	**EMFCC 0042**	**submerged wood**	**China**	**PP151720**	**PP153475**	**PQ578289**	**PQ601343**	**PQ601346**
***T. changchunensis***	**EMFCC 0046**	**submerged wood**	**China**	**PV082156**	**PV082158**	**PV082159**	**PV094898**	**PV094899**
***T. chiangmaiensis***	**KUMCC 16-0039**	***Chromolaena odorata***	**Thailand**	**MN061342**	**KY197856**	**KY197863**	**KY197876**	-
***T. chinensis***	**UESTCC 22.0085**	**submerged wood**	**China**	**OQ127986**	**OQ128004**	**OQ127995**	-	-
***T. chromolaenae***	**KUMCC 16-0036**	***Chromolaena odorata***	**Thailand**	**MN061345**	**KY197860**	**KY197867**	**KY197880**	**KY197873**
***T. fici***	**CBS 595.96**	***Ficus religiosa***	**Cuba**	**KF443408**	**KF443385**	**KF443387**	**KF443402**	**KF443395**
*T. fici*	KUMCC 16-0038	*Chromolaena odorata*	Thailand	MN061341	KY197859	KY197866	KY197879	KY197872
*T. fici*	KUMCC 16-0035	*Chromolaena odorata*	Thailand	MN061340	KY197858	KY197865	KY197878	KY197871
***T. gaodangensis***	**MFLUCC 17-0234**	**submerged wood**	China	**MF034135**	**MF034133**	**MF034134**	-	-
***T. goaensis***	**NFCCL 4040**	**decaying wood**	**India**	**KY440969**	**KY440970**	-	-	-
*T. herbarum*	CPC 24414	*Phragmites australis*	Netherlands	KR873260	KR873288	-	-	-
***T. hollandica***	**CBS 220.69**	***Delphinium* sp.**	**Netherlands**	**KF443406**	**KF443384**	**KF443389**	**KF443401**	**KF443393**
***T. hydei***	**KUMCC 16-0037**	***Chromolaena odorata***	**Thailand**	**MN061346**	**MH253926**	**MH253928**	**MH253930**	-
***T. lancangjiangensis***	**HKAS 112709**	**submerged wood**	**China**	**MW723059**	**MW879526**	**MW774582**	**MZ567104**	**MW729780**
***T. longan***	**ZHKUCC 22**-**0121**	***Dimocarpus longan***	**China**	**OR194035**	**OR194027**	**OR194032**	**OR228537**	**OR228535**
*T. longan*	ZHKUCC 22-0122	*Dimocarpus longan*	China	OR194036	OR194028	OR194033	OR228538	OR228536
***T. longiconidiophora***	**UESTCC 22.0088**	**submerged wood**	**China**	**OQ127983**	**OQ128001**	**OQ127992**	**OQ158977**	**OQ158967**
*T. longiconidiophora*	UESTCC 22.0125	submerged wood	China	OQ127984	OQ128002	OQ127993	OQ158976	OQ158972
***T. luguhuensis***	**KUNCC 22-12427**	**submerged wood**	**China**	**OQ729758**	**OQ947766**	-	**OQ999004**	**OQ999002**
***T. mackenziei***	**CCMJ 13080**	***Xanthoceras sorbifolium***	**China**	**PQ584885**	**PQ584887**	**PQ578287**	**PQ601341**	**PQ601344**
***T. mackenziei***	**CCMJ 13081**	***Xanthoceras sorbifolium***	**China**	**PQ584886**	**PQ584888**	**PQ578288**	**PQ601342**	**PQ601345**
***T. mackenziei***	**MFLUCC 13-0839**	***Bidens pilosa***	**Thailand**	**MN061344**	**KY197861**	**KY197868**	**KY197881**	**KY197874**
***T. masonii***	**CBS 245.57**	***Brassi* sp.**	**England**	**KR873261**	**KR873289**	-	-	-
***T. phytolaccae***	**ZHKUCC 22-0107**	***Phytolacca acinosa***	**China**	**ON611796**	**ON611800**	**ON611798**	**ON660881**	**ON660879**
*T. phytolaccae*	ZHKUCC 22-0108	*Phytolacca acinosa*	China	ON611797	ON611799	ON611797	ON660880	ON660878
***T. pluriseptata***	**MFLUCC 14-0437**	***Clematis vitalba***	**Italy**	**MN061338**	**KY197855**	**KY197862**	**KY197875**	**KY197869**
*T. polyseptata*	MFLUCC 17-1495	*Chromolaena odorata*	Thailand	MT214382	MT214476	MT214427	MT235791	MT235830
***T. sichuanensis***	**UESTCC 22.0087**	**submerged wood**	**China**	**OQ127981**	**OQ127999**	**OQ127990**	-	-
***T. suae***	**CGMCC 3.24259**	**submerged wood**	**China**	**OP359406**	**OP359415**	**OP369300**	**OP471618**	**OP476730**
***T. submersa***	**UESTCC 22.0086**	**submerged wood**	**China**	**OQ127985**	**OQ128003**	**OQ127994**	OQ158978	**OQ158968**
***T. sundara***	**MFLU 21-0089**	**bamboo culms**	**Thailand**	**OM276824**	**OM287866**	-	-	-
***T. thailandica***	**GZCC 20-0011**	**decaying wood**	**Thailand**	**MN907426**	**MN907428**	**MN907427**	-	-

**Table 2:** Percentage of growth inhibition (PGI) in in vitro antagonistic activity of *Torula* species against two pathogenic fungi after 7 days at 25 °C. Data are means of percentage of growth inhibition ± standard deviation (SD). Different letters in the same column indicate statistically significant differences between values. The coefficients *a* and *b* indicate significant differences between the groups.

Species	% PGI	
	*Botrytis cinerea*	*Cladobotryum mycophilum*
*Torula changchunensis*	19.01 ± 0.0406a	67.18 ± 0.0169a
*Torula mackenziei*	10.20 ± 0.0182b	24.57 ± 0.0259b

**Table 3:** Checklist of *Torula* species recorded in China.

Species	Host	Distribution
*Torula aquatica*	submerged wood	China, Yunnan
*T. aquilariae*	*Aquilaria sinensis*	China, Yunnan
*T. calceiformis*	dead wood	China, Guizhou
*T. camporesii*	herbaceous litter	China, Yunnan
*T. canangae*	submerged wood	China, Yunnan
*T. changchunensis*	submerged wood	China, Jilin
*T. chinensis*	submerged wood	China, Sichuan
*T. fici*	submerged wood	China, Yunnan
*T. gaodangensis*	submerged wood, *Malus* sp.	China, Guizhou
*T. lancangjiangensis*	submerged wood	China, Yunnan
*T. longan*	*Dimocarpus longan*	China, Guangdong
*T. longiconidiophora*	submerged wood	China, Sichuan
*T. luguhuensis*	submerged wood	China, Yunnan
*T. mackenziei*	submerged wood, *Xanthoceras sorbifolium*	China, Jilin, Yunnan
*T. masonii*	submerged wood, *Artemisia carvifolia*	China, Yunnan
*T. phytolaccae*	*Phytolacca acinosa*	China, Yunnan
*T. sichuanensis*	submerged wood	China, Sichuan
*T. suae*	submerged wood	China, Yunnan
*T. sundara*	submerged wood	China, Yunnan
*T. submersa*	submerged wood	China, Sichuan, Yunnan

## Data Availability

The original contributions presented in this study are included in the article/Supplementary Material. Further inquiries can be directed to the corresponding author.

## Supplementary Materials

The following are available online at https://www.mdpi.com/article/10.3390/microorganisms13071459/s1: Figure S1: Phylogram of *Torula* generated from maximum likelihood analysis based on combined ITS, LSU, SSU, *tef*1-α, and *rpb*2 sequence data.
