# Supplementary figures and images for: New Species, New Record, and Antagonistic Potential of Torula (Torulaceae, Pleosporales) from Jilin Province, China
Source: Microorganisms. 2025 Jun 23;13(7):1459. doi: 10.3390/microorganisms13071459 (PMC12300790; doi:10.3390/microorganisms13071459)

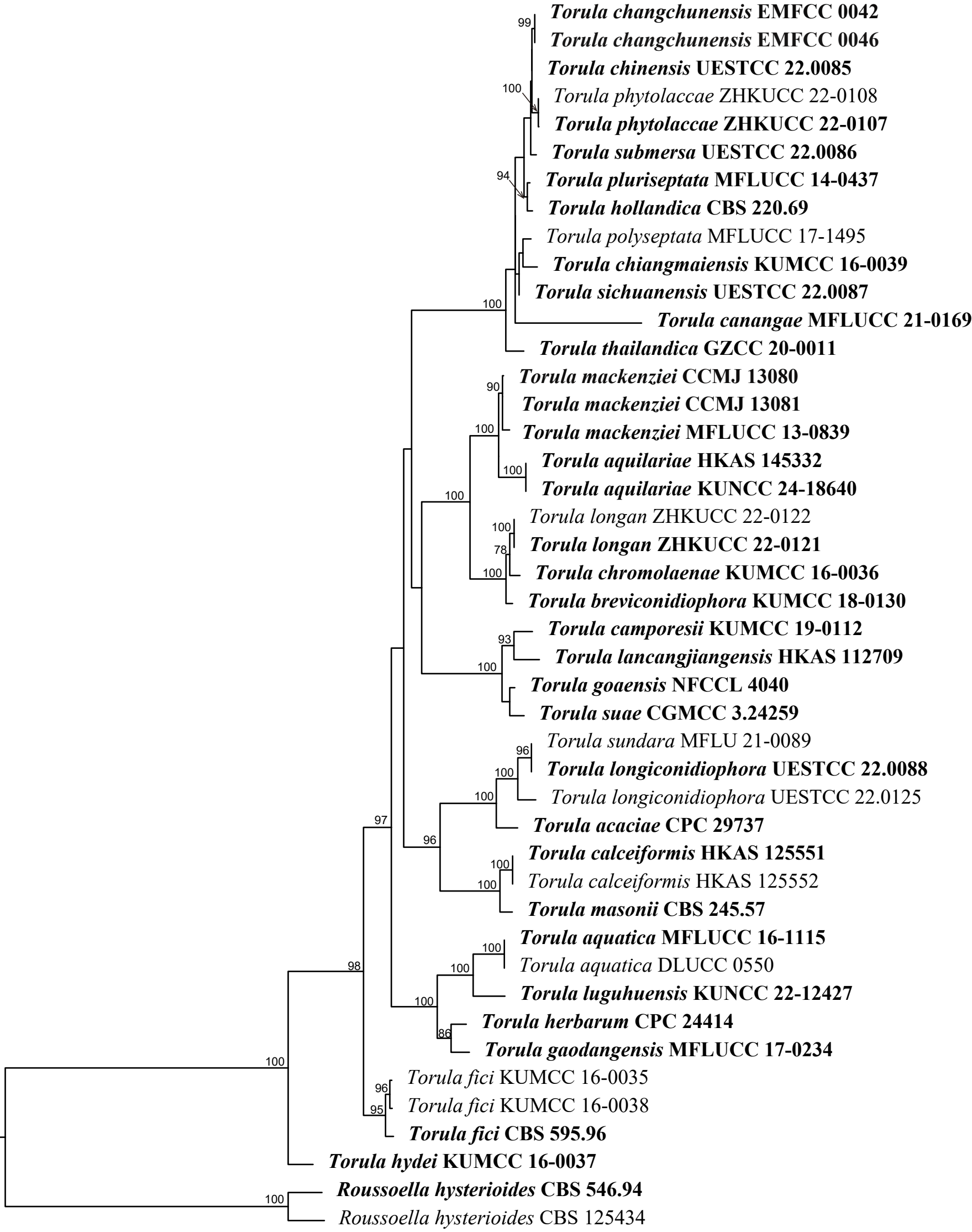

Supplement: Supplementary file 1 [file microorganisms-13-01459-s001.zip › microorganisms-3663813-supplementary.pdf]
